# The quality of reporting of pilot and feasibility cluster randomised trials: a systematic review

**DOI:** 10.1186/1745-6215-16-S2-P13

**Published:** 2015-11-16

**Authors:** Claire Coleman, Clémence Leyrat, Sandra Eldridge

**Affiliations:** 1 Queen Mary University of London, London, UK; 2 INSERM CIC 1415, CHRU de Tours, Tours, France

## The problem

A pilot or feasibility trial in preparation for a randomised trial of effectiveness is a study where part or all of a future trial is carried out on a smaller scale to see whether it can be done and whether we should proceed with it. We know reporting of pilot and feasibility studies is poor, and that these studies are particularly important when designing cluster randomised trials (CRT), in which clusters, rather than individuals, are the unit of randomisation. There are no previous reviews of the reporting quality of pilot and feasibility CRTs.

## The approach

We will systematically identify reports of pilot and feasibility CRTs published in English between 2011 and 2014. We expect around 40 studies. An electronic PubMed search, hand-search in online journals, and individual contacts will be used to identify papers. Quality assessment criteria will be based on the CONSORT extension for CRTs, and a CONSORT extension for pilot and feasibility randomised trials in the final stages of development.

## Findings

This project is in progress and will be completed by September 2015. Research to date has found quality of pilot and feasibility studies is poor so we expect that here. However, there have previously been no formal guidelines against which to assess quality of pilot and feasibility studies.

## Consequences

We will present recommendations for improving the conduct, analysis and reporting of these studies and expect this to improve future quality.

